# Altering visual feedback during reaching: no mitigating effect on pain for individuals with complex regional pain syndrome, but visuomotor adaptation is preserved

**DOI:** 10.3389/fnhum.2025.1701633

**Published:** 2025-12-17

**Authors:** Marion Dagenais, Chloé Sutter, Clémentine Brun, Anne Marie Pinard, Jean-Sébastien Roy, Catherine Mercier

**Affiliations:** 1 Center for Interdisciplinary Research in Rehabilitation and Social Integration (Cirris), Quebec City, QC, Canada; 2 Faculty of Medicine, Université Laval, Quebec City, QC, Canada; 3 CHU de Québec-Université Laval, Quebec City, QC, Canada

**Keywords:** complex regional pain syndrome, reaching, sensorimotor integration, visual feedback, visuomotor adaptation

## Abstract

**Introduction:**

Complex regional pain syndrome (CRPS) is often associated with pain-related fear of movement, and virtual reality has been proposed as a potential rehabilitation intervention to overcome this issue. Therefore, this cross-sectional study aimed to test whether altering the visual feedback (VF) on movement could mitigate pain and promote movement.

**Methods:**

Fifteen participants with upper-limb CRPS and 15 age- and gender-matched pain-free participants undertook a target reaching task in the Kinarm exoskeleton, with two VF conditions being tested (Per Exposure: GREATER or SMALLER VF; two separate sessions), preceded and followed by reaching movements without VF (Pre-/Post-Exposure). Proprioception was assessed with a Limb Position Sense task, and a Movement Accuracy outcome was derived from the Pre-Exposure reaching movements.

**Results:**

Contrary to our hypothesis, pain intensity was not influenced by VF conditions but increased over Time (*p* < 0.001). Analyses performed on kinematics data showed that participants displayed visuomotor adaptation both Per Exposure, and Pre-/Post-Exposure to altered VF (VF condition^*^Time: *p* < 0.001). Per Exposure analyses revealed that CRPS participants tended to adapt their Movement Length to a lesser extent than pain-free participants (Group^*^VF condition: *p* = 0.048). Pre-/Post-Exposure analyses revealed that CRPS participants consistently performed larger movements than pain-free participants (*p* = 0.002). Both groups performed similarly for the Limb Position Sense task, but CRPS participants displayed significantly larger errors for Movement Accuracy, suggesting impaired proprioceptive integration in the CRPS group.

**Discussion:**

These findings support the idea that visuomotor adaptation is preserved in CRPS and can be used to promote movement.

## 1 Introduction

Complex regional pain syndrome (CRPS) is a rare but debilitating chronic primary pain condition (Ferraro et al., 2024; Treede et al., 2019) that can lead to permanent functional impairments (Johnson et al., 2022; Lyckegård et al., 2024). CRPS is characterized by disproportionate pain, body perception disturbances, and sensorimotor disturbances (Ferraro et al., 2024; Brun et al., 2018a; Lewis et al., 2007). Previous work suggests that these sensorimotor disturbances could reflect impairments of both afferent (e.g., proprioceptive input) (Bank et al., 2013) and efferent (e.g., sensorimotor integration) nature in the treatment of sensorimotor information (Brun et al., 2020; Bultitude and Petrini, 2021).

Clinical guidelines for the management of CRPS emphasize the need for early interventions that encourage active limb movement and focus on restoring normal function; timely implementation of such interventions is essential to achieve these goals (Goebel et al., 2018; Harden et al., 2022; Harnik et al., 2023). However, CRPS, like other chronic pain conditions, is often associated with pain-related fear of movement (i.e., kinesiophobia), which leads to fear-avoidance behaviors (Farzad et al., 2024, 2022). Such avoidance behaviors can impede physical rehabilitation and recovery (Vlaeyen et al., 1995).

Over the last few decades, virtual reality interventions have been shown to be beneficial for chronic pain patients, serving as a distraction (Jones et al., 2016) and also mitigating pain while promoting movement (Harvie et al., 2015; Matamala-Gomez et al., 2019) [see (Viderman et al., 2023) for an umbrella review]. In a study involving participants with chronic neck pain, altering visual feedback (VF) from the visual scene during neck rotation movements was shown to have an immediate effect on pain, mitigating both intensity and onset. Specifically, pain intensity was lower and pain occurred later in the rotation in the “understated” VF condition, while pain intensity was higher and pain occurred earlier in the “overstated” VF condition (Harvie et al., 2015). Previous research in individuals with chronic upper-limb pain also showed that modifying VF of the size of the arm during hand movements had a sustained impact on pain and swelling, with the “magnified” VF condition being associated with the most pain and swelling, and the “minified” VF condition being associated with the least pain and swelling (Moseley et al., 2008). These examples suggest that manipulating the VF using virtual reality might decrease movement-associated pain during motor training and promote the execution of larger movements. However, no study so far has manipulated VF on the amplitude of limb movement in CRPS.

Thus, the general aim of this study was to assess whether altering the VF on limb movement could mitigate pain and promote movement in participants with upper-limb CRPS. The specific aims were twofold: to determine whether altering VF on movement (i.e., making it appear GREATER or SMALLER than the actual movement) could mitigate pain in participants with upper-limb CRPS (aim 1), and to compare movement adaptation to altered VF in adults with upper-limb CRPS compared to pain-free adults (aim 2). In this type of visuomotor adaptation paradigm, movement adaptation is expected to occur in the same direction as the manipulation Per Exposure to altered VF. Conversely, after-effects (Pre-/Post-Exposure effects) are expected to be in the opposite direction of the manipulation (Dagenais et al., 2021). Based on the study described above (Harvie et al., 2015), we hypothesized that pain intensity would be greater during the GREATER VF condition compared to the SMALLER VF condition (H1) because greater movements would be perceived as more threatening compared to smaller movements. We also hypothesized that CRPS participants would show decreased movement adaptation compared to pain-free participants during exposure to altered VF (H2.1), as well as decreased after-effects (H2.2). These hypotheses are based on previous work showing that individuals with chronic pain have more difficulty detecting VF manipulations than pain-free individuals (Brun et al., 2018b; Roosink et al., 2015). Moreover, CRPS can lead to proprioceptive deficits (Bank et al., 2013) and body perception disturbances (Lewis and Schweinhardt, 2012; Förderreuther et al., 2004), all of which can impede sensorimotor adaptation. Finally, a secondary aim was to compare proprioception between the two groups (aim 3).

## 2 Materials and methods

### 2.1 Participants and ethics statement

Fifteen participants with unilateral upper-limb (UL) CRPS, and 15 age- and self-reported gender-matched pain-free control participants were recruited between 2016 and 2024. The sample sizes reflect our recruitment capacity for CRPS participants during that period. Inclusion criteria for the CRPS group were: adults (≥18 years old) with a confirmed diagnosis of CRPS. Participants with CRPS were diagnosed in accordance with the Budapest clinical criteria (Goebel et al., 2018) by an anesthesiologist at the Center of Expertise in Chronic Pain in Quebec (AMP). Inclusion criteria for the control group were: pain-free adults (≥18 years old) without musculoskeletal disorder affecting the upper limbs and without any pain on the day of testing. Exclusion criteria for both groups were: motor, visual, or neurological impairments that could impede task completion. Participants in the CRPS group were recruited from the Center of Expertise in Chronic Pain, Quebec City (Qc, Canada), while control participants were recruited through community advertisements.

All participants provided written informed consent prior to their enrollment. The study adhered to the principles outlined in the Declaration of Helsinki, and the research protocol received approval from the local ethical review board (CIUSSS de la Capitale Nationale, Quebec City, Qc, Canada, no 2014-395).

### 2.2 Study design

**Figure 1:**
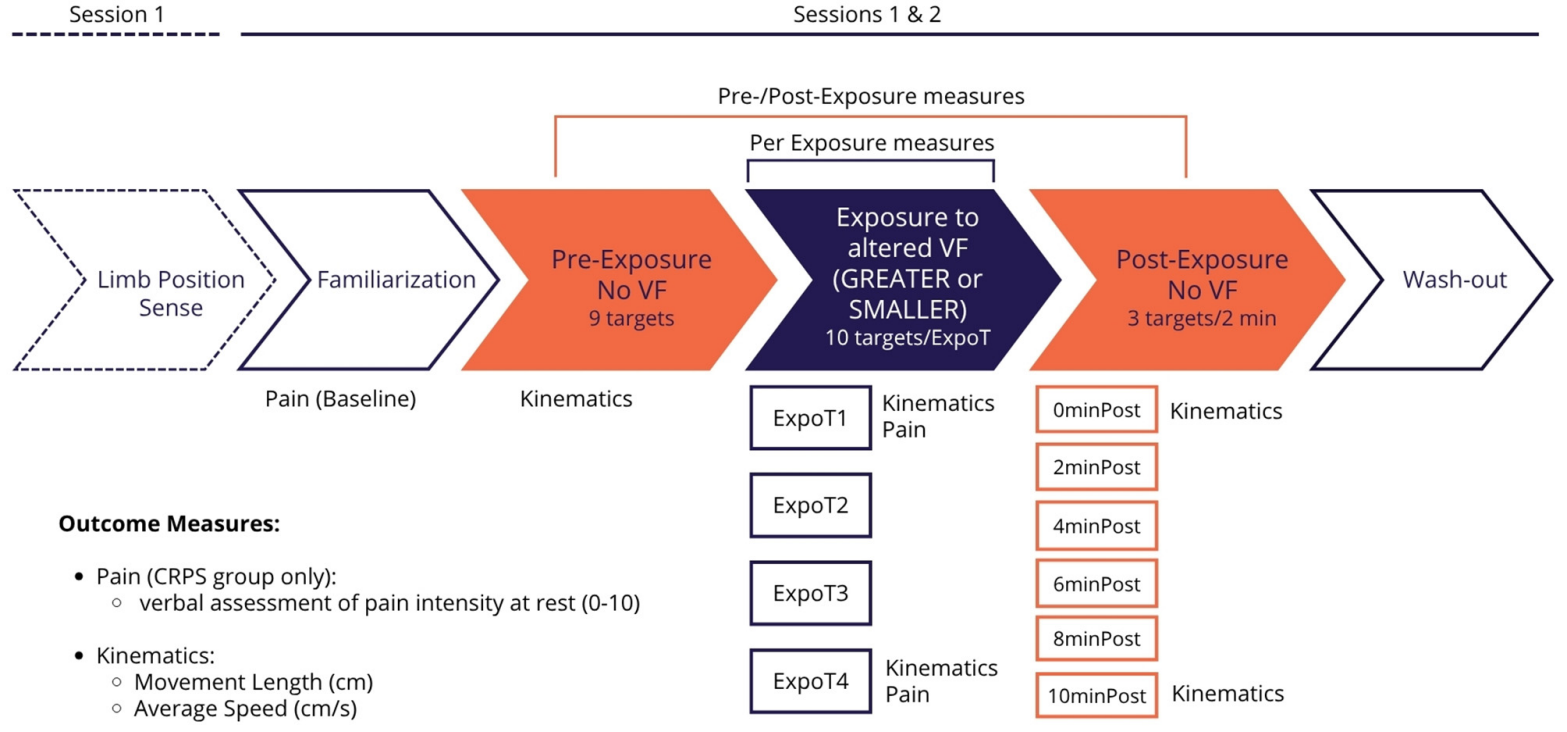
Study design. Session 1 began with the Arm Position Matching task. Sessions 1 and 2 comprised: the Familiarization task (6 targets, veridical VF), the Pre-Exposure task (9 targets, no VF), the Exposure to altered VF task (10 targets x 4 blocks, GREATER or SMALLER VF), the Post-Exposure task (3 of the 9 initial targets pseudo-randomly displayed every 2 min over a 10-min period, no VF), and the Wash-out task (6 targets, veridical VF). Kinematics measures (Movement Length and Average Speed) were acquired during Pre-Exposure, Exposure (ExpoT1 and ExpoT4), and Post-Exposure (0minPost and 10minPost) blocks. Pain intensity was assessed at rest with a verbal numerical rating scale ranging from 0 to 10 at three timepoints: after the Familiarization task (Baseline), after ExpoT1, and after ExpoT4.

The study was conducted in two sessions, separated by an average of 8.5 ± 4.4 days. At the beginning of the first session, participants' sociodemographic information and clinical characteristics of participants with CRPS (i.e., etiology, pain intensity, kinesiophobia) were collected through a semi-structured interview and the use of a self-reported questionnaire (see section below for details). The remainder of the two sessions was identical for both groups and consisted of a proprioceptive task (session 1 only) and a visuomotor adaptation task (sessions 1 and 2). Participants' ULs were concealed from view for both tasks. For the visuomotor adaptation task, participants reached a series of virtual targets with their affected UL (CRPS group) or with their dominant UL (control group). Different types of VF on movement were provided in real-time via a virtual UL superimposed to participants' actual UL: veridical VF (familiarization and wash-out), GREATER or SMALLER VF (Per Exposure to altered VF), or no VF (Pre-/Post-Exposure sequences). The GREATER and SMALLER VF conditions were each tested in a different session in a counterbalanced order across participants to control for order bias. See Figure 1 for the details about the course of experimental sessions.

### 2.3 Materials and procedure

#### 2.3.1 Kinarm Exoskeleton

**Figure 2:**
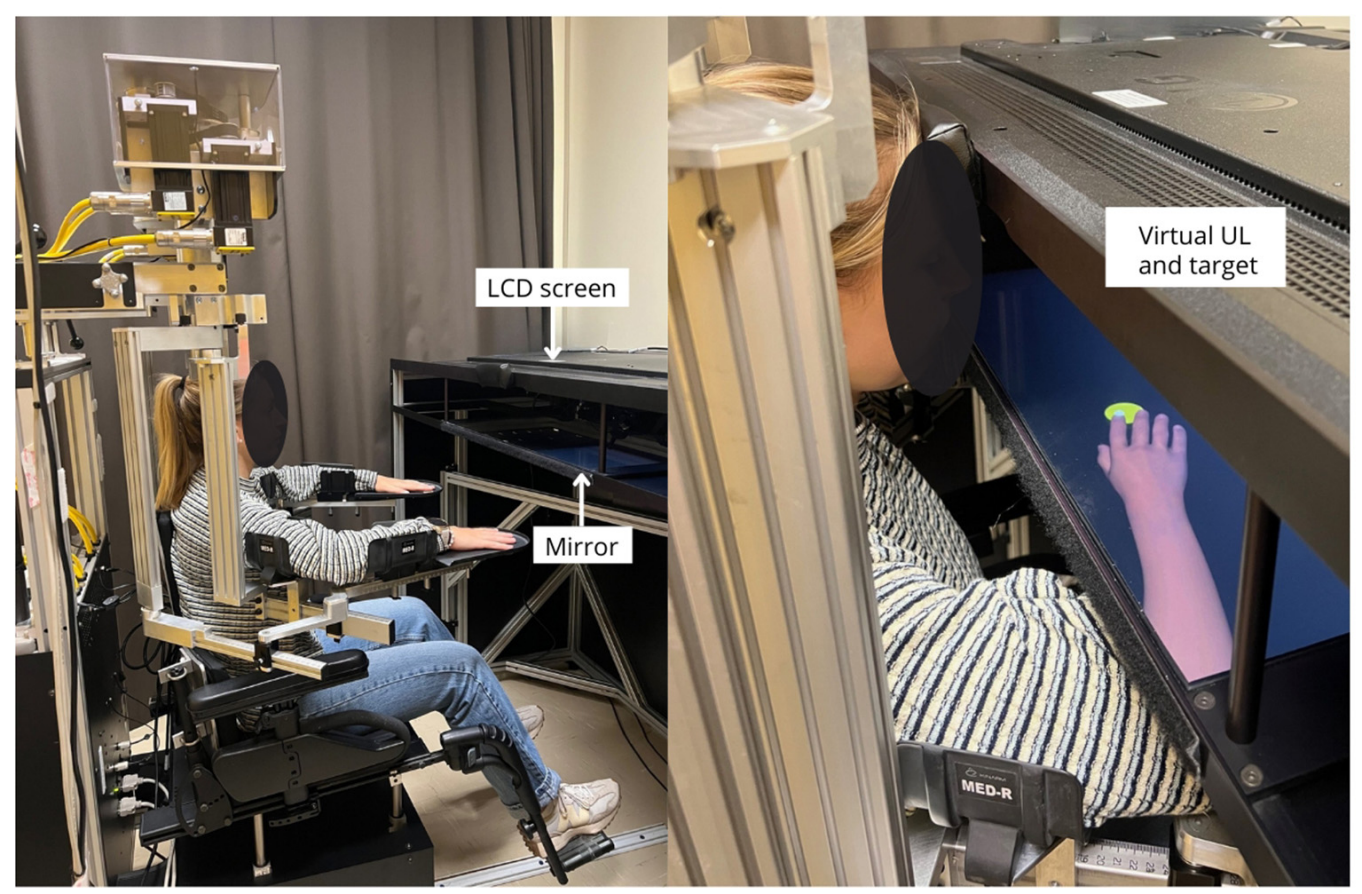
Experimental set-up. Kinarm system and virtual upper-limb (UL). The mirror located under the LCD screen creates a sense of depth so that the virtual UL appears to be located where the participant's real arm is, thus promoting an immersive experience. During the experiment, participants wore a bib (not shown in the picture) which occluded participants' ULs from view.

The proprioceptive and visuomotor adaptation tasks were performed using the Kinarm Exoskeleton Lab™ (Kinarm, Kingston, ON, Canada). The Kinarm Exoskeleton Lab is a bilateral robotic exoskeleton that allows passive or active control of participant movements at the shoulder (horizontal abduction-adduction) and elbow (flexion–extension) joints in the horizontal plane. Angles of the shoulder and elbow joints were recorded in real-time with the Kinarm motor encoders at a sampling rate of 1 kHz, allowing for real-time calculation of the index finger's position (see Figure 2). This setup provides a controlled environment for analyzing sensory and motor functions.

The altered VF was achieved by first adjusting the distance of the finger position from the starting landmark according to the selected scaling factor. In the GREATER VF condition, movements were displayed with an enlarged scaling factor of 1.5x, whereas in the SMALLER VF condition, the scaling factor was reduced to 0.67x. This means that the starting position was always appearing in its veridical location, while the target to be reached looked either farther or closer than their veridical location. Embedded functions in the software were employed to compute the corresponding shoulder and elbow joints angles, which were used to present a virtual arm sized for each participant according to the scaling factor as described in a previous study from our lab (Dagenais et al., 2021).

#### 2.3.2 Self-reported questionnaire

Kinesiophobia was assessed with the Tampa Scale for Kinesiophobia (TSK-11). The TSK-11 is a self-reported questionnaire developed to assess pain-related and injury-related fear of movement in individuals with chronic musculoskeletal pain (Kori et al., 1990). The French-Canadian version of the TSK-11 was used in the present study (French et al., 2002).

#### 2.3.3 Limb Position Sense (session 1)

For the first session, participants were required to complete the Kinarm Standard Arm Position Matching Task (Dukelow et al., 2010). In this task, no VF was provided. The robot passively positioned the affected limb (CRPS group)/non-dominant limb (control group) into one of four predefined positions. Participants then had to actively mirror-match the position with their unaffected limb (CRPS group)/dominant limb (control group). A total of 24 trials were executed. This approach has been successful to assess proprioception in prior studies focusing on assessing clinical populations such as stroke survivors (Dukelow et al., 2010) and individuals with chronic pain (Brun et al., 2018a, 2020; Dagenais et al., 2021).

#### 2.3.4 Adaptation to altered visual feedback during a target reaching task (sessions 1 and 2)

**Figure 3:**
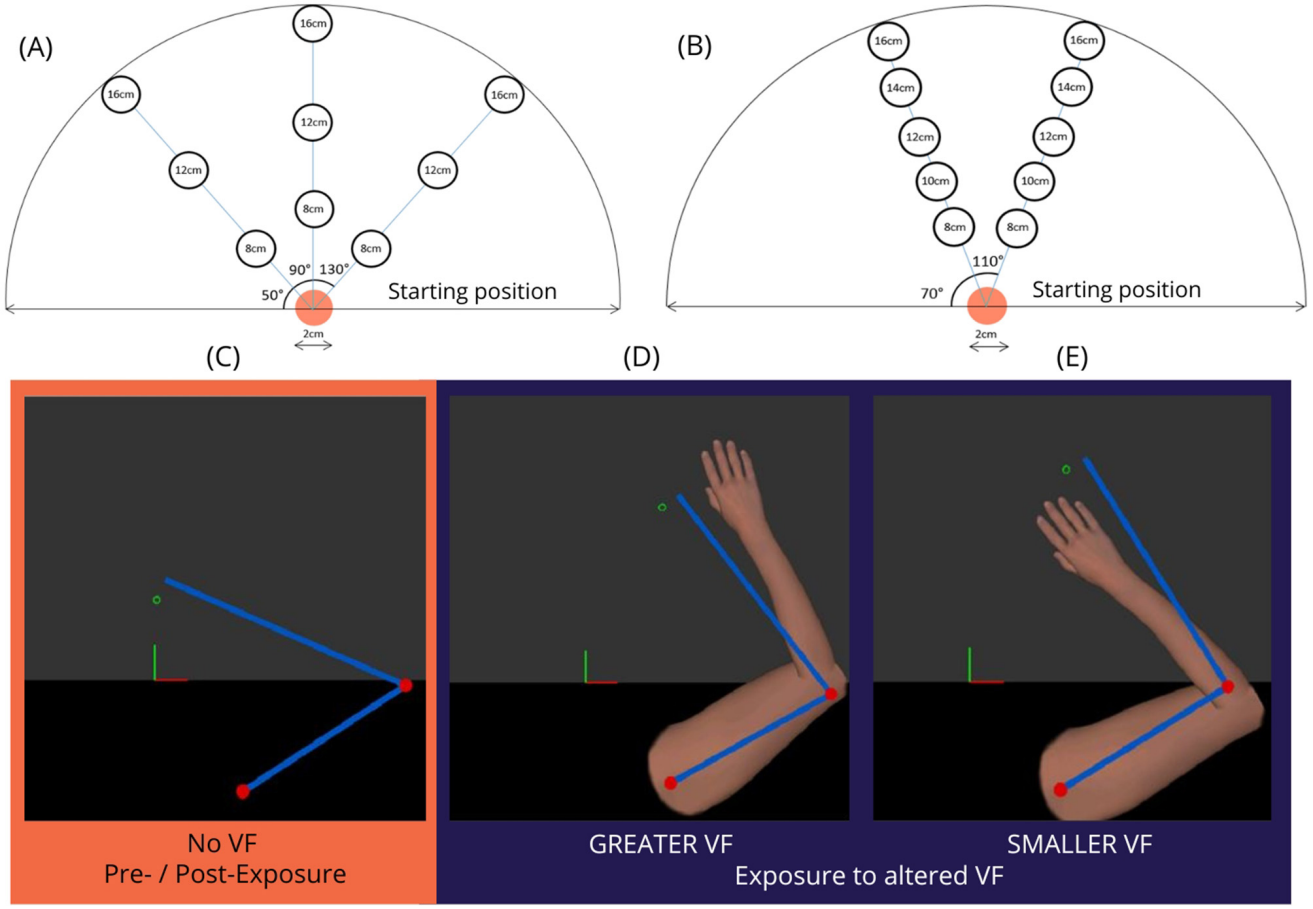
Upper panel: Target location for the **(A)** Pre- and Post-Exposure tasks and **(B)** Per Exposure to altered visual feedback (VF). Lower panel: types of VF: **(C)** no VF on arm location was provided during the Pre- and Post-Exposure blocks, while during Exposure to altered VF, the virtual upper-limb displayed GREATER **(D)** or SMALLER **(E)** VF on movement compared to participant's actual movements. The blue bars represent the participant's arm and forearm location, while the red dots and green empty circle represent the participant's shoulder, elbow, and index fingertip, respectively. These markers (bars, dots and circle) were only visible to the experimenter.

The visuomotor adaptation task comprised a series of target reaching movements performed with participants' self-reported dominant UL (control group) or CRPS-affected limb (CRPS group). A virtual UL replacing the actual UL displayed either a GREATER or SMALLER VF on movement. These sequences of altered VF were framed by a period of familiarization at the beginning and washout at the end. Reaching movements without VF were also performed before (Pre-) and after (Post-) exposure to the altered VF to test for after-effects. Participants were asked to perform their movements as accurately as possible, at a comfortable speed. Figure 3 shows the details about target positions (panels A, B) and VF conditions (panels C, D, E) for the reaching tasks.

##### 2.3.4.1 Familiarization and wash out

In these sequences, participants saw a virtual UL corresponding to the veridical VF of their movement, and they had to perform six reaches toward virtual targets. This was done to help participants become familiar (familiarization) with the exoskeleton and the reaching task. The task was repeated at the end of each session (wash-out) to reduce any potential carry-over adaptation effects from the altered VF.

##### 2.3.4.2 Pre- and Post-exposure sequences

For these sequences, no VF was provided (see Figure 3C). At the beginning of the task, the robot passively moved the participants' UL to the starting position. At this position, a visual signal indicated they were in the correct position, and they had to remain there for 2 s before the target appeared. Participants were then asked to actively reach for these targets with the index fingertip. Targets were located either on the median line, or ±40° from the median line, and at 8, 12 or 16 cm from the starting position, averaging 12 ± 4 cm in required movement length (see Figure 3A). Participants had to indicate verbally to the experimenter when they thought they were in the center of the target before returning to the starting position. The same nine targets were used before (Pre-) and after (Post-) exposure to altered VF, with a difference in the temporality of the presentations (randomized order). Before exposure, the nine targets were presented randomly. After exposure, the same targets were displayed in blocks of three targets. These blocks were presented in a randomized order every 2 min, over a 10-min period. To assess whether adaptation to altered VF could generalize to different movements, target positions were not the same as Per Exposure to altered VF.

##### 2.3.4.3 Per Exposure to altered visual feedback

Participants performed goal-directed reaching movements under visual guidance of a virtual UL that displayed either GREATER or SMALLER VF on movement (see Figures 3D, E). Each of the four blocks of trials consisted of reaching in a pseudo-random order to one of ten 2 cm diameter targets located at ±20° from the midline, requiring a reaching movement of 8, 10, 12, 14 or 16 cm from the starting position, averaging 12 ± 4 cm in required movement length (see Figure 3B).

At the beginning of each of the ten trials per block (four blocks in total), the robot passively moved the UL to the starting position. For each of the ten trials, the starting position was shown to the participant for 2 s before one of the ten targets appeared. Participants were instructed to actively reach the target with the index fingertip and to maintain the final position for 2 s. After this lapse of time, the starting position reappeared and the participant moved back to the starting position, waiting for the next target to appear.

### 2.4 Outcome measures

#### 2.4.1 Primary outcome measures

##### 2.4.1.1 Pain intensity

Pain intensity of CRPS participants was assessed with a verbal numerical rating scale (vNRS) ranging from 0 (no pain) to 10 (worst possible pain) at rest at three different time points of each experimental session: after the Familiarization task (Baseline), after the first exposure block (ExpoT1), and after the fourth exposure block (ExpoT4) (see Figure 1 for details).

##### 2.4.1.2 Movement Kinematics

For each trial, the x, y coordinates of the fingertip position were computed in real-time by the Dexterit-E software. Kinematic variables were then calculated offline using custom-made MATLAB 2015 scripts (Mathworks inc., Massachusetts). A vector between the starting position and the position where the movement stopped (i.e., when movement speed was < 2 cm/s) was calculated, constituting the **Movement Length** (cm). This outcome was chosen to focus mainly on the feedforward component of movement control (i.e., by removing the small corrections at the end of the reaching movement). **Average Speed** (cm/s) was computed for the same interval as the Movement Length.

##### 2.4.1.3 Alteration detection

To assess explicit awareness of the alteration, participants were required to report which VF condition they had received at the end of each session. Reported responses were “GREATER VF,” “SMALLER VF” or “Don't know”. It is worth noting that, at the beginning of each session, participants were informed that VF would either be GREATER or SMALLER. It was unknown to them whether VF condition would vary with or be the same between sessions.

#### 2.4.2 Secondary outcome measures

##### 2.4.2.1 Proprioception

Two different measures of proprioception were used: the Limb Position Sense, which is a classical position matching task, and a measure of Movement Accuracy derived from the Pre-Exposure measure of the visuomotor adaptation task.

The z-score of the task-score (z-task score) obtained for the Kinarm Arm Position Matching task was used as a measure of Limb Position Sense, and to assess for proprioceptive deficits in participants. A z-task score of ≥1.65 indicated that participants' performance was poorer than normative data for age-, sex- and dominance-matched controls (Simmatis et al., 2020).

For Movement Accuracy, we calculated the vector (in cm) between the end of movement point (speed < 2 cm/s) and the target coordinates during the Pre-Exposure trials (i.e., reaching toward a target in the absence of VF) of the visuomotor adaptation task. Although this variable mostly reflects the accuracy of the motor prediction, it depends on the updating of internal models, which also reflects how proprioceptive information is integrated to optimize movement (Scott, 2004).

### 2.5 Statistical analyses

Mean ± standard deviation is reported in the results. The Mann-Whitney U test was used to compare age between groups. Non-parametric analyses for longitudinal data (nparLD) were used to assess pain modulation and movement adaptation. NparLD analyses are well indicated for small sample sizes and for data that is not normally distributed (Noguchi et al., 2012).

#### 2.5.1 Aim 1: pain modulation

Pain intensity was analyzed using a 2 [VF condition (GREATER/SMALLER)] x3 [Time (Baseline/ExpoT1/ExpoT4)] nparLD analysis. *Post hoc* analyses were performed using Wilcoxon tests with Benjamini-Yekutieli correction for the p-values.

#### 2.5.2 Aim 2.1: visuomotor adaptation Per Exposure to altered VF

Movement Length and Average Speed were analyzed using 2 [VF condition (GREATER/SMALLER)] x2 [Time (ExpoT1/ExpoT4)] x2 [Group (CRPS/control (CTRL))] nparLD analyses. *Post hoc* analyses were performed using Wilcoxon tests (intra-group measures) and Mann-Whitney U tests (inter-group measures) with Benjamini-Yekutieli correction for the p-values.

##### 2.5.2.1 Alteration detection

A chi-square test of independence was performed to determine whether the proportion of participants who accurately detected both VF conditions differed across groups.

#### 2.5.3 Aim 2.2: visuomotor adaptation Pre-/Post-exposure to altered VF

Movement Length and Average Speed were analyzed using 2 [VF condition (GREATER/SMALLER)] x3 [Time (Pre-Exposure/0minPost-Exposure/10minPost-Exposure)] x2 [Group (CRPS/CTRL)] nparLD analyses. *Post hoc* analyses were performed using Wilcoxon tests (intra-group comparisons) and Mann-Whitney U tests (inter-group comparisons) with Benjamini-Yekutieli correction for the *p*-values.

#### 2.5.4 Aim 3: proprioception

Limb Position Sense and Movement Accuracy were compared between groups using Mann-Whitney U tests. Spearman correlation coefficients were used to assess the relationship between the two proprioception variables.

NparLD analyses were performed with R (version 4.4.1) and all other analyses were performed with IBM SPSS Statistics software version 29 (IBM Corp., Armonk, New York). Significance was set at *p* < 0.05.

## 3 Results

Thirty participants took part in this study, including 15 individuals with upper-limb CRPS (13 women, mean age 56.5 ± 10.6 years) and 15 pain-free participants (13 women, mean age 54.3 ± 9.2 years) matched for gender and age. All participants completed the entire study, therefore there were no missing data. CRPS participants' demographic and clinical characteristics are reported in Table 1. There was no age difference between the two groups (U = 131.0, n1 = 15, n2 = 15, *p* = 0.46).

**Table 1:** Demographic and clinical characteristics of CRPS participants.

**CRPSXX**	**Gender**	**Age (years)**	**Handedness**	**Affected side**	**Etiology**	**Time since diagnosis (months)**	**Pain (NRS)**	**TSK-11**
01	W	62	Right	D	Wrist sprain	6	4	27
02	W	61	Right	D	Wrist surgery	20	5	30
03	M	49	Left	D	Hand fracture	4	7	32
04	W	59	Right	D	Hand fracture + surgery	4	0.5	23
05	W	50	Right	ND	Wrist surgery	1	1	26
06	W	54	Right	ND	Hand fracture + surgery	5	2	30
07	W	60	Right	ND	Wrist sprain	168	6	28
08	W	60	Right	D	Wrist fracture	1	7	28
09	W	62	Right	ND	Wrist fracture + surgery	1	2.5	21
10	W	63	Right	ND	Wrist fracture + surgery	2	0	14
11	W	26	Right	D	Glass splinter in 5^th^ digit + abscess	6	6	19
12	W	47	Right	ND	Hand fracture	2	2	32
13	W	69	Right	ND	Wrist fracture + surgery	1	2.5	25
14	W	57	Right	D	Hand fracture	16	2	17
15	M	69	Right	D	Wrist fracture	2	2.5	22
Mean ± SD		56.5 ± 10.6				15.9 ± 42.4	3.3 ± 2.3	24.9 ± 5.5

W, Woman; M, Man; D, dominant; ND, non-dominant; Pain (NRS), current pain intensity assessed at rest with a verbal numerical rating scale; TSK-11, kinesiophobia assessed with the Tampa Scale for Kinesiophobia - 11-item version.

### 3.1 Aim 1: pain modulation

**Figure 4:**
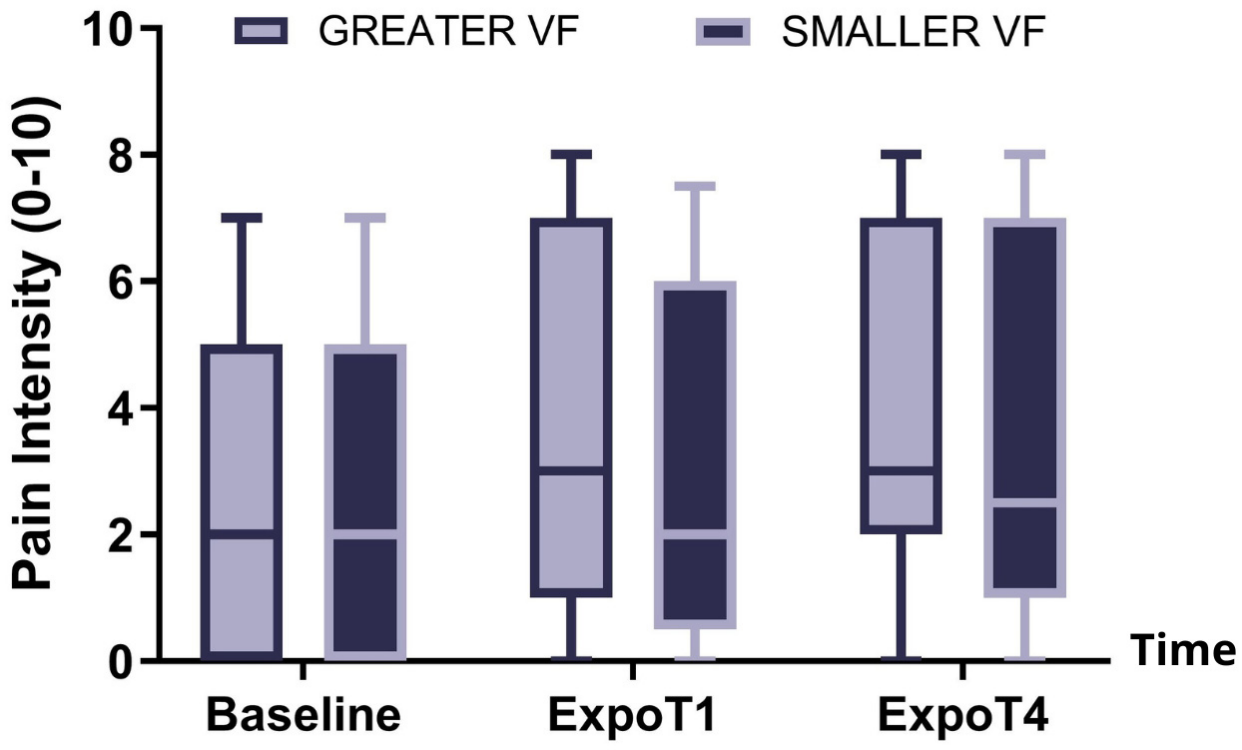
Pain intensity at Baseline, ExpoT1, and ExpoT4 for the GREATER (light background, dark outline) and SMALLER (dark background, light outline) visual feedback conditions. The box represents the interquartile range, and the line crossing the box represents the median value. Error bars represent the minimal and maximal values.

There was no main effect of the VF condition on pain intensity (*p* = 0.65), nor a significant interaction between Time and VF condition (*p* = 0.13) (see Figure 4). There was a main effect of Time on pain intensity (*p* < 0.001). *Post hoc* analyses revealed significant differences in pain intensity between each time point (all *p* < 0.05), with an increase observed throughout the course of the sessions.

### 3.2 Aim 2.1: visuomotor adaptation Per Exposure to altered VF

**Figure 5:**
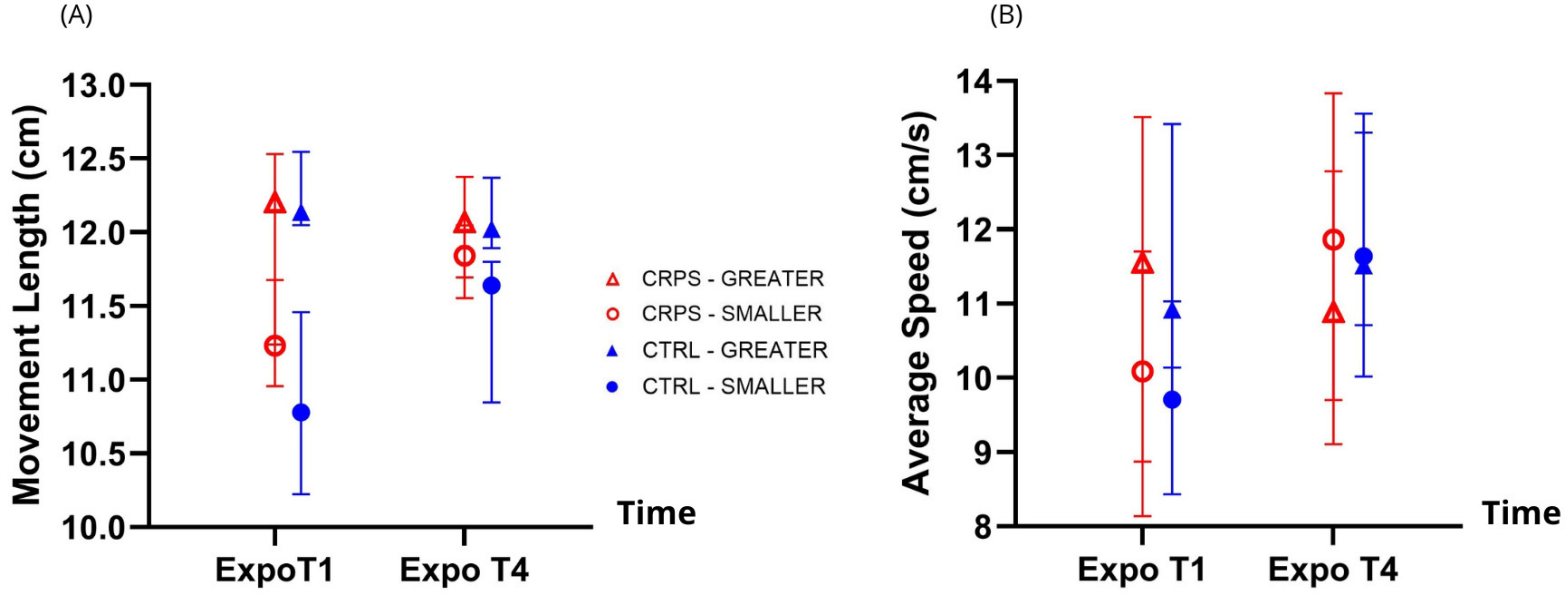
Adaptation Per Exposure to the two altered visual feedback (VF) conditions across groups. As expected, participants adapted their Movement Length **(A)** and Average Speed **(B)** in a way that is consistent with the displayed VF condition. There were also differences in adaptation patterns to the two VF conditions between groups for Movement Length, with a tendency for CRPS participants to display less movement adaptation compared to CTRL participants, however these differences were not significant. This tendency was not observed for Average Speed. The triangles and circles represent the median values. Error bars represent the interquartile range.

Figure 5 shows Per Exposure adaptation to the two altered VF conditions across groups, for both Movement Length (panel A) and Average Speed (panel B). Since the hypotheses primarily focus on interaction effects, these results will be presented in the text, but not the main effects. All *p*-values for Aim 2.1 (main effects and interactions) are presented in Table 2.

**Table 2:** *P*-values of the nparLD analyses for movement adaptation (movement length and average speed) Per Exposure to altered VF.

**Aim 2.1: Per Exposure**		**Movement length**	**Average speed**
		***p*** **-value**	***p*** **-value**
Main effects	Group	0.77	0.67
	VF condition	**< 0.001**	0.30
	Time	**0.002**	**< 0.001**
Simple interactions	Time^*^VF condition	**< 0.001**	**< 0.001**
	- ExpoT1: SMALLER < GREATER^*^	**< 0.001** ^*^	0.06
	- ExpoT4: SMALLER < GREATER^*^	**< 0.001** ^*^	0.82
	- SMALLER: ExpoT1 < ExpoT4	**0.004**	**< 0.001**
	- GREATER: ExpoT1 = ExpoT4	0.72	1.0
	Group^*^VF condition	**0.048**	0.77
	- CRPS: SMALLER < GREATER	**0.002**	
	- CTRL: SMALLER < GREATER	**< 0.001**	
	- SMALLER: CRPS = CTRL	0.21	
	- GREATER: CRPS = CTRL	1.0	
	Group^*^Time	0.26	0.38
Double interaction	Group^*^VF condition^*^Time	0.76	0.52

VF, visual feedback; CRPS, complex regional pain syndrome; CTRL, control. *P*-values in bold indicate statistically significant effects. Significant effects for either Movement Length or Average Speed (but not both variables) are identified with an asterisk (*).

#### 3.2.1 Movement length

There was a significant Time^*^VF condition interaction (*p* < 0.001), indicating that participants adapted their Movement Length in a way that is consistent with the VF condition (i.e., expected effects: target undershooting in the SMALLER VF condition, and target overshooting in the GREATER VF condition, with an attenuation of these differences over the course of exposure). Indeed, *post hoc* analyses revealed that Movement Length was smaller for the SMALLER VF condition compared to the GREATER VF condition, both at ExpoT1 and ExpoT4 (both *p* < 0.001), and that Movement Length was smaller at the beginning of exposure (ExpoT1) to SMALLER VF than at the end of exposure (ExpoT4) (*p* = 0.004). However, movement length was not different between ExpoT1 and ExpoT4 for the GREATER VF condition.

There was a significant Group^*^VF condition interaction (*p* = 0.048), indicating that groups behaved slightly differently when exposed to the VF conditions. Both groups displayed smaller movements in the SMALLER VF condition compared to the GREATER VF condition (*p* = 0.002 for the CRPS group and < 0.001 for the CTRL group). However, *post hoc* analyses looking at group differences for each VF condition were not statistically significant.

The Group^*^Time simple interaction and the Group^*^VF condition^*^Time double interaction were not statistically significant.

#### 3.2.2 Average speed

There was a significant Time^*^VF condition interaction (*p* < 0.001), indicating that participants adapted their movement speed in a way that is consistent with the VF condition (i.e., expected effects: slower movements in the SMALLER VF condition, and faster movements in the GREATER VF condition, with an attenuation of these differences over the course of exposure). *Post hoc* analyses revealed that reaching movements were slower at ExpoT1 compared to ExpoT4 in the SMALLER VF condition (*p* < 0.001), but no other differences were found.

There were no other significant simple nor double interactions for Average Speed.

#### 3.2.3 Alteration detection

Only 13% (*n* = 2) of participants in the CRPS group were able to accurately identify both VF conditions, compared to 53% (*n* = 8) of participants in the CTRL group. The chi-square test of independence showed that the proportion of accurate detection was significantly different between groups [X^2^ (1, *N* = 30) = 5.40, *p* = 0.020].

### 3.3 Aim 2.2: visuomotor adaptation Pre-/Post-exposure to altered VF

**Figure 6:**
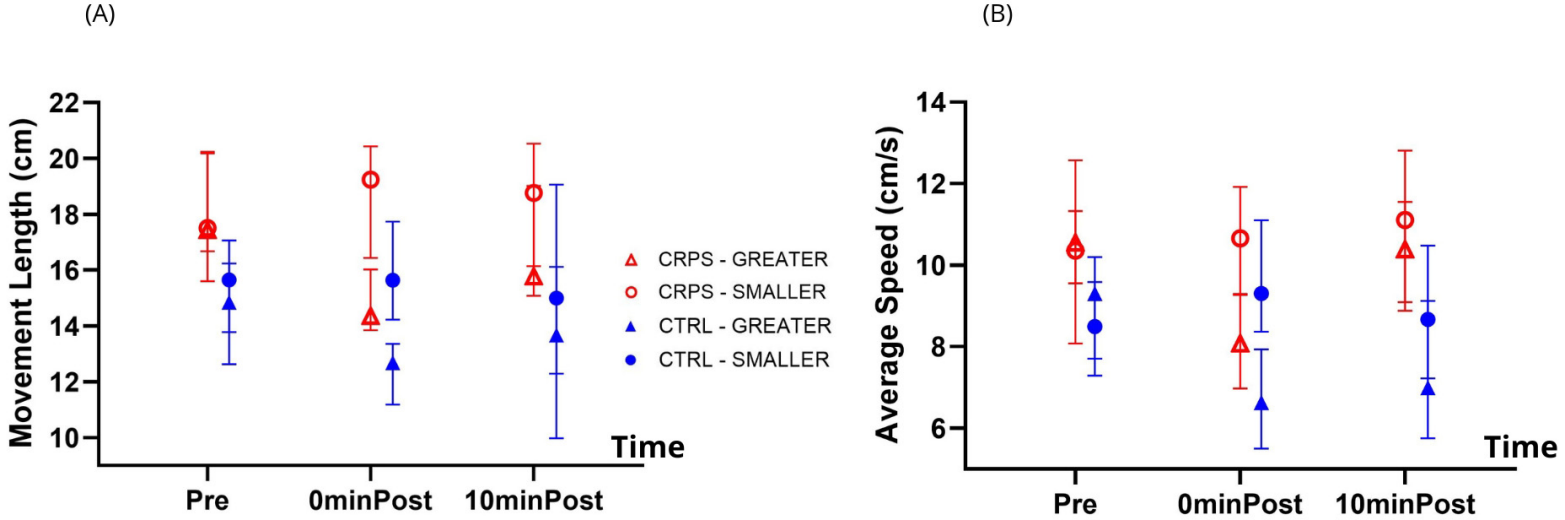
Adaptation to the two visual feedback (VF) conditions: After-effects across groups. As expected, participants adapted their Movement Length **(A)** and Average Speed **(B)** in a way that is consistent with the displayed VF condition. There were also differences in the time course of adaptation between the two groups for Movement Length, and this effect was mainly driven by greater Movement Length for CRPS participants at every timepoint. This tendency was not observed for Average Speed. The triangles and circles represent the median values. Error bars represent the interquartile range.

Figure 6 shows After-effects across groups for Movement Length (panel A) and Average Speed (panel B).

Since the hypotheses primarily focus on interaction effects, these results will be presented in the text, but not the main effects. All *p*-values for Aim 2.2 are presented in Table 3 (main effects and interactions).

**Table 3:** *P*-values of the nparLD analyses for movement adaptation (movement length and average speed) Pre-/Post-exposure to altered VF (after-effects).

**Aim 2.2: Pre-/post-exposure**		**Movement length**	**Average speed**
		***p*** **-value**	***p*** **-value**
Main effects	Group	**0.002**	**0.004**
	VF condition	**< 0.001**	**< 0.001**
	Time	**< 0.001**	**0.022**
Simple interactions	Time^*^VF condition	**< 0.001**	**< 0.001**
	- Pre: SMALLER = GREATER	1.0	0.17
	−0minPost: SMALLER > GREATER	**< 0.001**	**< 0.001**
	−10minPost: SMALLER > GREATER	**< 0.001**	**0.036**
	- SMALLER: Pre < 0minPost^*^	0.063	**0.036** ^*^
	- SMALLER: Pre = 10minPost	1.0	0.66
	- SMALLER: 0minPost = 10minPost	0.49	0.96
	- GREATER: Pre > 0minPost	**< 0.001**	**< 0.001**
	- GREATER: Pre > 10minPost	**0.001**	0.064
	- GREATER: 0minPost < 10minPost^*^	0.10	**0.036** ^*^
	Group^*^Time	**0.029**	**0.026**
	- Pre: CRPS > CTRL^*^	**0.029** ^*^	0.26
	−0minPost: CRPS > CTRL^*^	**0.029** ^*^	0.45
	−10minPost: CRPS > CTRL^*^	**0.029** ^*^	0.06
	- CRPS: Pre > 0minPost^*^	**0.029** ^*^	0.20
	- CRPS: Pre = 10minPost	0.40	1.0
	- CRPS: 0minPost = 10minPost	0.36	0.26
	- HC: Pre = 0minPost	0.07	0.26
	- HC: Pre > 10minPost	**0.029** ^*^	0.26
	- HC: 0minPost = 10minPost	0.49	1.0
	Group^*^VF condition	0.95	0.46
Double interaction	Group^*^VF condition^*^Time	0.82	0.52

VF, visual feedback; CRPS, complex regional pain syndrome; CTRL, control. *P*-values in bold indicate statistically significant effects. Significant effects for either Movement Length or Average Speed (but not both variables) are identified with an asterisk (*).

#### 3.3.1 Movement length

There was a significant Time^*^VF condition interaction (*p* < 0.001), indicating that participants adapted their Movement Length in a way that is consistent with the VF condition (i.e., expected after-effects: larger movements Post-Exposure to SMALLER VF, and smaller movements Post-Exposure to GREATER VF). Indeed, *post hoc* analyses revealed that Movement Length was not different between VF conditions in the Pre-Exposure block, but that movements were larger in the SMALLER VF condition compared to the GREATER VF condition, both at 0minPost-Exposure and 10minPost-Exposure (both *p* < 0.001). *Post hoc* analyses looking at time comparisons did not reveal any significant differences in Movement Length in the SMALLER VF condition, while Movement Length in the GREATER VF condition was larger in the Pre-Exposure block compared to 0minPost-Exposure (*p* < 0.001) and 10minPost-Exposure (*p* = 0.001), but did not differ between 0minPost-Exposure and 10minPost-Exposure.

There was a significant Group^*^Time interaction (*p* = 0.029), indicating that the two groups displayed a slightly different time-course of adaptation. *Post hoc* analyses revealed that CRPS participants produced larger movements than CTRL participants at every time point (all *p* = 0.029), which is also supported by a main effect of Group (*p* = 0.002). *Post hoc* analyses looking at time comparisons within each group showed that CRPS participants displayed larger movements in the Pre-Exposure block compared to 0minPost-Exposure (*p* = 0.029), and that CTRL participants displayed larger movements in the Pre-Exposure block compared to 10minPost-Exposure (*p* = 0.029). No other differences were found.

The Group^*^VF condition simple interaction and the Group^*^VF condition^*^Time double interaction were not statistically significant.

#### 3.3.2 Average speed

There was a significant Time^*^VF condition interaction (*p* < 0.001), indicating that participants adapted their movement speed in a way that is consistent with the VF condition (i.e., expected after-effects: faster movements Post-Exposure to SMALLER VF, and slower movements Post-Exposure to GREATER VF). *Post hoc* analyses comparing VF conditions at different time points showed that while the Average Speed did not differ between VF conditions during the Pre-Exposure block, movements were faster at 0minPost-Exposure and 10minPost-Exposure to SMALLER VF (*p* < 0.001 and 0.008, respectively). *Post hoc* analyses looking at time differences within each VF condition showed that movements were slower in the Pre-Exposure compared to 0minPost-Exposure to SMALLER VF (*p* = 0.008), but there were no other differences between time points for this VF condition. For the GREATER VF condition, movements were faster in the Pre-Exposure block compared to 0minPost-Exposure (*p* < 0.001) and 10minPost-Exposure (*p* = 0.040), the movements being slowest at 0minPost-Exposure (*p* = 0.010).

There was a significant Group^*^Time interaction (*p* = 0.029), indicating that the two groups displayed a slightly different time-course of adaptation. However, none of the *post hoc* comparisons were statistically significant.

The Group^*^VF condition simple interaction and the Group^*^VF condition^*^Time double interaction were not statistically significant.

### 3.4 Aim 3: proprioception

#### 3.4.1 Limb Position Sense

**Figure 7:**
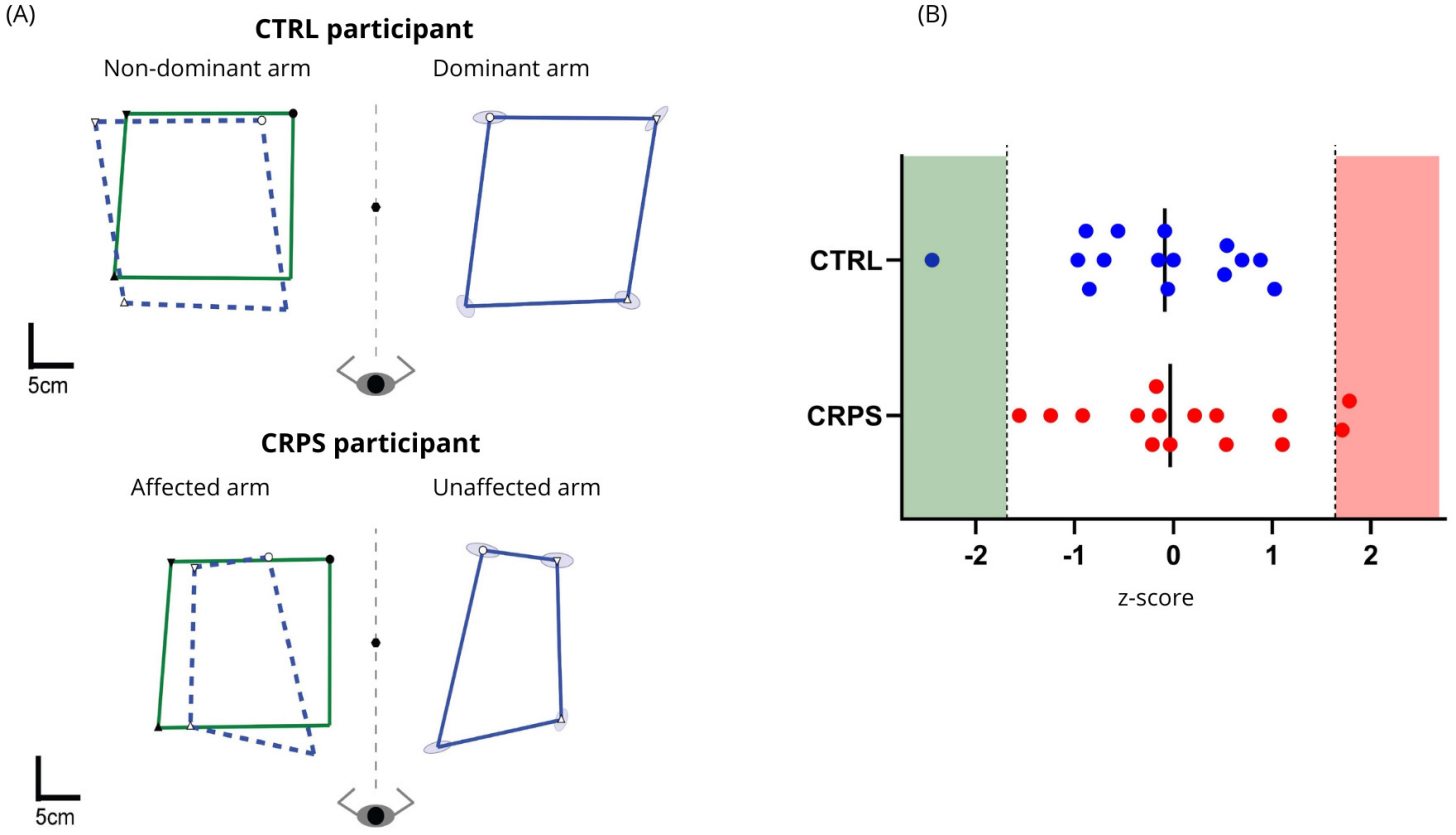
Arm Position Matching Task. **(A)** Individual data of representative participants from each group. The green squares represent the movements performed by the robot (i.e., passive movements of the affected/non-dominant arm), while the blue squares represent the movements performed by the participants (i.e., active movements of the unaffected/dominant arm). The blue dashed squares are a superimposition of participants' matched movements compared to the movements performed by the robot. **(B)** Performance distribution (z-scores) for both groups. Values comprised between −1.65 and 1.65 represent a performance within the normative range, while values inferior to −1.65 indicate a better-than-average performance (green section), and values greater than 1.65 indicate a poorer-than-average performance (red section).

Although two CRPS participants performed worse than the normative data and one CTRL participant performed better than the normative data, overall, participants‘ performance on the Arm Position Matching task did not differ significantly between the two groups (CRPS: mean z-task score = 0.15 ± 0.99; CTRL: mean z-task score = −0.20 ± 0.91) (U = 129.0, n1 = 15, n2 = 15, *p* = 0.51). Figure 7A shows an example of representative participants from each group. Figure 7B shows participants' z-task score distribution.

#### 3.4.2 Movement accuracy

**Figure 8:**
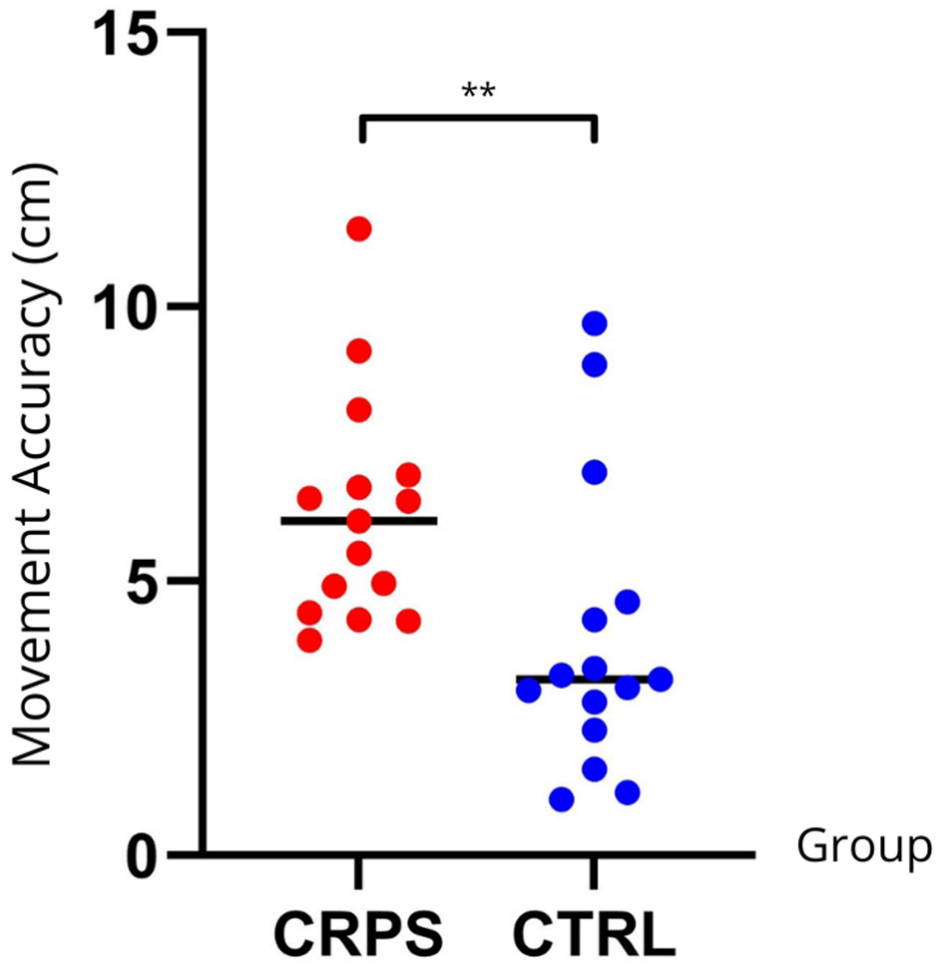
Individual data distribution for Movement Accuracy across groups. Larger values indicate larger errors between the end of movement and the target to reach. CRPS participants performed significantly larger errors compared to CTRL participants. Significant effects for either Movement Length or Average Speed (but not both variables) are identified with an asterisk (*).

CRPS participants displayed significantly larger errors during the Pre-Exposure blocks compared to CTRL participants (CRPS: 5.75 ± 1.98 cm; CTRL: 3.60 ± 2.28 cm) (U = 180, n1 = 15, n2 = 15, *p* = 0.004) (see Figure 8).

Since Movement Accuracy was different across groups, two separate Spearman coefficients were calculated to assess the relationship between Limb Position Sense and Movement Accuracy. There were no significant correlations between the two variables, neither for the CRPS group nor for the CTRL group (CRPS: rho = 0.10, *p* = 0.72; CTRL: rho = 0.4, *p* = 0.14).

## 4 Discussion

The general aim of this study was to assess whether altering the VF on movement could influence pain and movement in participants with upper-limb CRPS. Overall, our results show that altering the VF on movement did not modulate pain, and that both CRPS and CTRL participants adapted their movements to altered VF, with only slight differences between groups. CRPS participants had significantly more difficulty to accurately detect the VF conditions, despite showing preserved visuomotor adaptation. Finally, CRPS participants displayed larger errors (Movement Accuracy) than CTRL participants during reaching movements without VF (Pre-Exposure trials). However, there were no significant proprioceptive deficits during the Limb Position Sense task, and the two proprioception variables were not related.

Contrary to our hypothesis (H1), pain increased over the course of experimental sessions, but no differences were observed between the two conditions of altered VF in terms of the evolution of pain ratings during the task. Several reasons could explain this result. First, the increase in pain ratings might be attributable to the sheer duration of the task and repetitive use of the affected limb, which is irrespective of the VF condition. Moreover, the absence of a modulating effect of altered VF on pain ratings could be explained by CRPS participants' difficulty in accurately detecting the VF conditions. This observed difficulty in detecting VF alterations is aligned with previous findings in chronic pain populations (Brun et al., 2018a; Dagenais et al., 2021; Roosink et al., 2015). Since our hypothesis relied on the reasoning that larger movements would appear more threatening than smaller movements, the fact that most participants were unable to differentiate between the two VF conditions (alteration detection: only 13% of CRPS participants accurately identified both VF conditions) could have prevented this mitigating effect. One could question whether VF manipulations were simply too subtle to be detected by participants. However, the scaling factor used in the present study (1.5x) was equivalent to, or even greater than that used in other VF manipulation studies (Harvie et al., 2015; Dagenais et al., 2021; Roosink et al., 2015), which supports our methodological choice. On the other hand, one cannot exclude that the inherent cognitive load of pain and other CRPS symptoms had an impact on VF manipulation detection. Indeed, previous work has found that some individuals with CRPS showed impaired cognitive processing (see Halicka et al., 2020 for a review). Nevertheless, there is mitigated evidence regarding the role of explicit detection of VF manipulation on pain modulation altogether. Indeed, in a recent study investigating the effect of VF manipulation by ±10% of lumbar amplitude during lumbar extension movements with chronic low-back pain participants, the authors found that movement-evoked pain occurred later in the movement for the understated condition, despite participants having not noticed the VF manipulation. The authors also found that participants with higher levels of kinesiophobia (i.e., average TKS-11 score of 33.6 ± 5.3) responded better to the VF manipulation (Jordán-López et al., 2025). On that note, it is worth mentioning that TSK-11 scores were not particularly high in our sample (24.9 ± 5.5, the possible range for this questionnaire being 11–44). Other studies conducted with CRPS participants reported higher scores on the TSK-11 (Farzad et al., 2024, 2022; Lewis et al., 2024), which could have prevented us from seeing a modulating effect of the VF conditions on pain ratings. However, this hypothesis remains to be tested. Lastly, similar results were obtained in a previous study performed with fibromyalgia participants in our lab using the same methodology and experimental set-up as the present study (Dagenais et al., 2021). It is also worth noting that previous work comparing the modulating effect of different VF conditions on pain has obtained diverging results, despite having used similar methodologies (Harvie et al., 2015; Kragting et al., 2020).

Regarding visuomotor adaptation, our results show that both groups adapted their movements in a way that is consistent with the VF conditions, but that CRPS participants displayed adaptation patterns that differed slightly from those observed in CTRL participants. While visuomotor adaptation was observed for both Movement Length and Average Speed, the results were more pronounced for Movement Length. As such, the following discussion will focus on this outcome.

Our results show that CRPS participants adapted their movements Per Exposure to altered VF, but that adaptation patterns to the VF conditions differed slightly compared to that of CTRL participants, which supports our hypothesis (H2.1). In fact, CRPS participants showed a tendency to adapt their movements to a lesser extent (i.e., less difference in Movement Length between the two VF conditions at ExpoT1) than CTRL participants, but the differences observed did not reach significance in the *post hoc* analyses. These results are similar to those reported by Vittersø and collaborators, who compared visuomotor adaptation to prism goggles between CRPS participants and pain-free participants, as well as between affected/non-dominant and non-affected/dominant arms. They observed similar endpoint errors during prism exposure between the two groups, thus concluding that visuomotor adaptation is preserved in persons with CRPS (Vittersø et al., 2021). This is also consistent with the results of previous studies—including our study with fibromyalgia participants (Dagenais et al., 2021)—suggesting that visuomotor adaptation is preserved in individuals with chronic pain (Chen et al., 2017, 2014).

Our results also show that participants displayed after-effects that are consistent with the VF conditions (significant Time^*^VF condition interaction), which again is consistent with previous findings supporting preserved visuomotor adaptation in CRPS (Vittersø et al., 2021) and supports our hypothesis (H2.2). However, the time course of adaptation differed slightly between the two groups, which mainly manifested as significantly larger movements in the CRPS group. In fact, CRPS participants systematically produced larger movements than CTRL participants, including during the Pre-Exposure block (CRPS: 18.16 ± 2.15 cm; CTRL: 15.50 ± 3.13 cm), suggesting that these differences are not driven by differences in visuomotor adaptation, but rather by impaired pointing accuracy. CRPS participants made larger pointing errors when visual information about limb position was unavailable—notably, such differences were not observed during exposure to altered VF, i.e., in the presence of VF on movement. These results are consistent with previous findings showing that individuals with CRPS make larger pointing errors than pain-free individuals when visual information about limb position is unavailable (Verfaille et al., 2021; Lewis et al., 2010). One explanation for this phenomenon is that individuals with CRPS may have impaired integration of proprioceptive information (Bultitude and Petrini, 2021; Lewis et al., 2010), which would prevent the optimal use of proprioceptive information to update internal models, therefore impeding the production of precise movements (Frith et al., 2000). Also supporting this explanation is a study by Bank and collaborators that highlighted the presence of proprioceptive impairments not only in the CRPS-affected hand, but also in the unaffected hand, therefore suggesting impaired central processing (Bank et al., 2013). Finally, altered proprioceptive integration—rather than impaired registration and perception of proprioceptive afferent information—would explain why participants in the present study produced significantly larger movements (and greater errors for the Movement Accuracy outcome) than control participants during the Pre-/Post-Exposure blocks, despite having shown a similar performance in the Limb Position Sense task (aim 3).

### 4.1 Limitations

This study has limitations that should be carefully considered when interpreting and generalizing the results. First, it is worth mentioning that participants in the CRPS group displayed mild-to-moderate levels of impairments [notably, pain intensity at rest was 3.3 ± 2.3 in our sample, which is lower than other studies (Schulte-Goecking et al., 2020; Halicka et al., 2024; Acapo et al., 2025)]. This could be explained by the fact that they were receiving active care at the Center of Expertise in Chronic Pain at the time of recruitment. Also, since inclusion relied on participants having sufficient motor function and tolerance to be able to complete the reaching task, it possibly precluded more severely impaired individuals from taking part in the present study. We acknowledge that this limits the external validity of our sample and prompts caution when interpreting our results. Moreover, to prevent attrition due to pain and fatigue in the CRPS group, methodological decisions were made to limit the burden of the experiment. As such, our experimental design only comprised two altered VF conditions, and no veridical VF condition. This limits the interpretation of the modulating effect of altering the VF on movement, since there is no control condition to compare the changes in pain ratings over the course of experimental sessions. Furthermore, we only tested the participants' affected arm, as opposed to both the affected and unaffected arm, which precludes comparisons of visuomotor adaptation patterns between the two arms, which also limits the interpretation of our results.

## 5 Conclusion

In conclusion, our results suggest that visuomotor adaptation is preserved in individuals with CRPS, despite the presence of impaired proprioceptive integration (i.e., errors in Movement Accuray in the absence of VF) and impaired detection of VF manipulations. As such, altering the VF on movement can promote movement adaptation, making it a potentially useful intervention with individuals with CRPS. As for pain modulation, the present study adds to previous work showing contradictory results regarding the potential of manipulating VF to modulate pain intensity. Thus, future work should aim to gain clarity about the mechanisms underlying pain mediation in individuals with CRPS.

## Data Availability

The raw data supporting the conclusions of this article will be made available by the authors, without undue reservation.
